# Foaming Behavior and Microcellular Morphologies of Incompatible SAN/CPE Blends with Supercritical Carbon Dioxide as a Physical Blowing Agent

**DOI:** 10.3390/polym11010089

**Published:** 2019-01-08

**Authors:** Hai-Chen Zhang, Chun-Na Yu, Yong Liang, Gui-Xiang Lin, Cong Meng

**Affiliations:** 1 School of Materials Science & Energy Engineering, Foshan University, Foshan 528000, China; hczhang@fosu.edu.cn (H.-C.Z.); lgx2272079639@163.com (G.-X.L.); 2 Guangzhou Quality Supervision and Testing Institute, Guangzhou 511447, China; 3 School of Mechanical and Vehicle Engineering, Changzhou Institute of Technology, Changzhou 213032, China; 4 Key Laboratory of Polymer Processing Engineering, Ministry of Education, South China University of Technology, Guangzhou 510640, China; mengcong629@163.com

**Keywords:** supercritical CO_2_ foaming, interconnected pores, bubble interface nucleation, blends

## Abstract

The foaming process and cellular morphologies of poly(styrene-co-acrylonitrile) (SAN)/chlorinated polyethylene (CPE) blends with supercritical carbon dioxide (scCO_2_) as a blowing agent were investigated in this study. As compared to pure SAN foam in the same batch, the foamed blends with various CPE elastomer content had smaller average pore size and larger cell density. This is probably related to the inhibition of bubble growth by elastomer, resulting in poor melt flowability and strong viscoelasticity, and the efficient bubble heterogeneous nucleation caused by numerous phase interfaces inside the incompletely compatible blend system. In addition, many tiny interconnected holes through the pore walls were formed to connect adjacent micropores in foamed blend samples. The formation mechanism of such interconnected pores is probably due to the fracture of stretched melt around the bubble from phase interfaces with weak interactions. These facts suggest an effective path to control pore size, cell density and even interconnected pores of blend foams depends on the compatibility of the blend system and difference in foamability of individual components in supercritical CO_2_.

## 1. Introduction

Microcellular polymeric foams are very attractive porous materials with wide application in the packaging, isolation and automotive industries because of their light weight, good heat or sound insulating properties, high impact strength and low cost [1,2,3,4]. Such foams have small cell diameter, ranging from 0.1 to 100 μm and cell density greater than 10^8^ cells/cm^3^, which gives them superior overall performance over traditional foams with chemical blowing agents [5,6,7]. Generally, most thermoplastic polymers can be foamed with hydrocarbons or fluorocarbons (CFCs) or reactive substances, but their foams often tend to have an average pore diameter of hundreds of microns and these types of blowing agents are not very suitable for the environment. Thus, many efforts are now being devoted to using more environmentally friendly physical blowing agents such as supercritical carbon dioxide (CO_2_) or nitrogen (N_2_) as substitutes.

**Figure 1:**
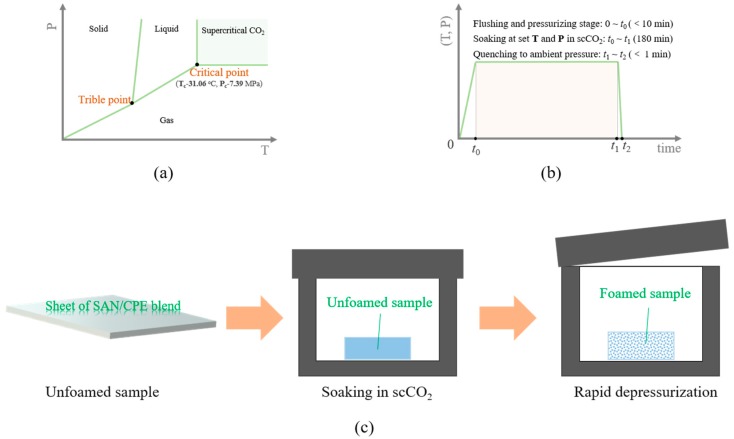
(**a**) Pressure-temperature phase diagram of supercritical CO_2_; (**b**) Schematic diagram of sample treatment and foaming process with supercritical fluid; (**c**) Illustration of one-step foaming process with supercritical carbon dioxide (scCO_2_) as a physical blowing agent.

Actually, supercritical carbon dioxide (scCO_2_) has been widely used as an efficient physical blowing agent to prepare microcellular polymeric foams, owing to its moderate supercritical condition (see Figure 1a), gas-like diffusivity and viscosity, liquid-like density and negligible surface tension [8,9,10,11]. In the supercritical state, CO_2_ molecules can quickly diffuse into the polymeric matrix to form a single-phase polymer/fluid solution and the materials can easily achieve higher equilibrium solubility at the end of the soaking process. When the foaming process is triggered by thermodynamic fluctuations that are generally caused by sudden depressurization or rapid heating, numerous CO_2_ molecules tend to aggregate and form a dense gaseous nucleus. After growth and solidification, they grow into a large number of pores in the matrix.

In contrast to classical foaming methods, porous materials with smaller pores and higher cell density can be easily obtained by the supercritical fluid foaming method and the microstructures or physical properties of the foams can also be effectively controlled by modulating the saturated conditions and foaming process. For instance, by increasing the solubility and reducing the foaming temperature close to the glass transition temperature (T_g_) of polymer matrix, the cell size of poly (methyl methacrylate) (PMMA) foams decreases from the micron to nanoscale, and the cell density reaches as high as 10^14^ cells/cm^3^, resulting in a significant improvement in the mechanical behavior at high strain rates as well as the shore hardness of foams [12,13,14,15,16]. Bimodal porous foams have also been fabricated using a simple two-step depressurization foaming process [17,18] so that simultaneously, the bimodal structures and porosity could be tuned by adjusting foaming parameters, including temperature, intermediate pressure, soaking time and holding time. In addition, functional foams with highly oriented porous structures or ordered porous layer structures can be manufactured by controlling the composition [19], surface constrained condition, crystallization or flow field. These advantages prompted us to choose this promising, environmentally friendly foaming approach.

Poly(styrene-co-acrylonitrile) (SAN) is an amorphous thermoplastic engineering plastic with excellent chemical stability and mechanical properties [20,21]. SAN resin has good processability and foamability similar to that of amorphous polystyrene (PS) and its foams are commonly used in sandwich sheets, especially in marine environments, and also as decorative interior moldings. Lee et al. successfully prepared microcellular SAN foams by using a rapid depressurization foaming process and carefully investigated the effects of several foaming parameters on their porous structures [22]. The average pore size increased and cell density decreased with increasing temperature, while higher CO_2_ pressure produced lower cell size but higher cell densities. Urbanczyk et al. studied the effects of nanoclay on the foaming process and foam morphologies of SAN/nanoclay nanocomposites in detail [23]. It was found that well dispersed nanoclay could greatly increase the bubble nucleation quantity and a lower foaming temperature (below the T_g_ of SAN resin) greatly increased the cell density of foams. Conceivably, the foamability and microstructures of SAN can also be regulated by blending it with other polymers. As previously described, microcellular SAN foams by supercritical CO_2_ foaming are usually closed cell structures.

In this study, thermoplastic chlorinated polyethylene (CPE) rubber was selected to blend with SAN resin and the blends were foamed via a batch foaming process with scCO_2_ as the environmentally benign blowing agent. The effects of thermoplastic rubber on melt processability, foamability and porous morphologies were carefully investigated. In particular, the formation process of interconnected pores in SAN/CPE blends and the corresponding pore opening mechanism resin was examined as well.

## 2. Materials and Methods

### 2.1. Materials

The commercial resin of styrene-acrylonitrile with 21% AN (SAN, BHF) employed in this work was provided in pellets form by China Petroleum Lanzhou Petrochemical Company. The chlorinated polyethylene (CPE, CM352) elastomer with 32% chlorine was purchased from Keli Chemical Co., Ltd. (Hangzhou, China). Commercial purity grade CO_2_ (99.9%) was obtained from Foshan Deli Gas Inc. (Foshan, China).

### 2.2. Preparation of SAN/CPE Blends

Blends of SAN and CPE were melt-blended with various weight ratio (90/10, 80/20, 70/30, 60/40, 50/50) at 190 °C for 15 min using a Haake Rheomix internal mixer (Thermo Fisher Scientific, Dresden, Germany). Prior to mixing, SAN and CPE pellets were dried for 4 h in a vacuum oven at 80 °C. The blends of SAN/CPE were compressed into 1mm specimens at 190 °C under 15 MPa for 5 min, for the following characterization and foaming experiments.

### 2.3. Foaming of SAN/CPE Blends with Supercritical CO_2_

The foams of SAN/CPE blends were obtained with a quenching method described by Geol and Beckman [7], namely, a one-step foaming process as depicted in Figure 1c. The plate-shaped samples were saturated with scCO_2_ in a self-made autoclave at a certain saturation pressure and temperature as described in Figure 1b. The foaming temperature ranged from 70 °C to 130 °C and the pressure was from 15 MPa to 25 MPa. Before equilibrating to saturating conditions, the vessel was flushed with low-pressure CO_2_ at room temperature for about 5 min to get rid of the air. After soaking for 3 h, the pressure was quickly quenched to ambient pressure (at an average speed of about 1 MPa/s) to obtain porous samples. Then, these samples were allowed to full growth at 70 °C and ambient pressure for 30 min before the vessel was cooled down to room temperature to maintain the porous structures. The volume expansion ratios of foamed samples (being inversely proportional to the apparent density of foams) were measured via water displacement [4,24].

### 2.4. Characterization

#### 2.4.1. Melt Flow Index (MFI)

The melt flow index (MFI) of composites was determined with an XNR-400 melt flow index instrument (Xiangli Instruments, Guangzhou, China) in accordance with the ASTM D1238-04 standard. The test temperature was 230 °C and the loading point was 5 kg. The MFI (g/10 min) value of composites was calculated with the formula as follow:

(1) $$\mathrm{MFI}=\frac{600m}{t}$$

where *m* is the average mass and *t* is the time required for each cut segment, their units are gram (g) and second (s), respectively.

#### 2.4.2. Thermogravimetric Analysis (TGA)

The thermal stability of the SAN/CPE composites was determined by a thermogravimetric analyzer (TG209, NETZSCH, Selb, Germany) at a heating rate of 10 °C /min under air atmosphere, from room temperature to 550 °C. Samples for testing were approximately 8 mg.

#### 2.4.3. Differential Scanning Calorimetry (DSC)

Differential scanning calorimetry (DSC) tests were carried out under nitrogen atmosphere at a heating and cooling rate of 10 °C/min to investigate the thermal properties of neat SAN, neat CPE and SAN/CPE blends with a NETZSCH DSC204 F1 analyzer (NETZSCH, Selb, Germany). All samples were first heated to 180 °C and kept isothermal for 5 min to eliminate thermal history, and then they were cooled to 30 °C and subsequently reheated from 30 °C to 180 °C at 10 °C/min. The glass transition temperature (T_g_) was determined from the second heating scan.

#### 2.4.4. Dynamic Thermomechanical Analysis (DMA)

The dynamic mechanical properties of SAN/CPE blend samples were determined using a TA DMA Q800 analyzer (TA Instruments Inc., New Castle, PA, USA) in extensional mode at a frequency of 1 Hz and a strain amplitude of 0.05%. Measurements of the storage modulus (E′), the loss modulus (E′′) and the dissipation factor (tan δ) were carried out in the temperature range of −50 °C to 140 °C at a heating rate of 5 °C/min.

#### 2.4.5. Field-Emission Scanning Electron Microscopy (FSEM)

Field-emission scanning electron microscopy (FSEM; FEI Inspect F, FEI Company, Hillsboro, Oregon, USA) was conducted to observe the fractured surface morphologies of composites and the porous structures of foams with 20 kV accelerating voltage. The unfoamed and foamed specimens were fractured to expose the internal structure after being immersed in liquid nitrogen for 30 min. Prior to morphology observation, the fractured surface was sputter-coated with gold to provide enhanced conductivity. Image analysis was performed on the SEM micrographs using Image J software to obtain the average cell size and cell density. The mean cell diameter (*D*) and cell density (*N_c_*) of the foamed samples were estimated by:

(2) $$D=\frac{\sum_{i=1}^{n}d_{i}}{n}$$

(3) $$N_{c}={\left[\frac{nM^{2}}{A}\right]}^{\frac{3}{2}}$$

where *n* is the number of cells in the micrograph, $d_{i}$ is the equivalent diameter of one cell, *M* is the magnification factor of the SEM image, and *A* (cm^2^) is the apparent area of the SEM micrograph.

## 3. Results and Discussion

### 3.1. Thermogravimetric Analysis and Melt Flow Index of SAN/CPE Blends

**Figure 2:**
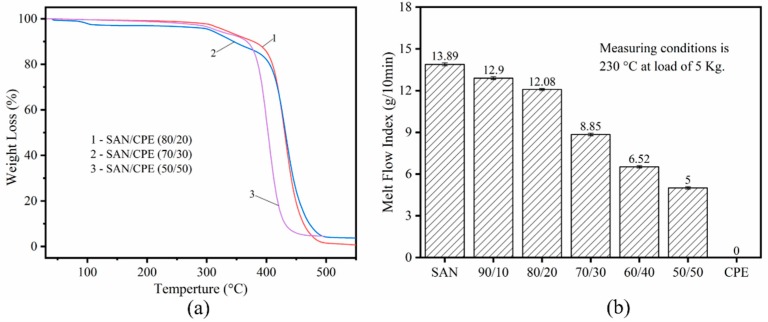
(**a**) TGA profile of SAN/CPE composites; (**b**) Melt flow index of SAN/CPE composites at 230 °C with 5 kg loadings.

The thermal stability of SAN/CPE composites was determined by thermogravimetric analysis. As can be seen from the scanning curves in Figure 2a, the initial decomposition temperature of blends with 20, 30 and 50 wt.% CPE content were about 312.9, 300.5 and 303.4 °C, respectively, which is much higher than the experimental temperature, implying that no significant thermal degradation occurs during the soaking stage and foaming process. 

It is well known that the foaming behavior of polymers is strongly dependent on their melt strength. Generally, foaming behavior is qualitatively analyzed by the melt flow index, which usually has an inverse relationship with the melt strength and a positive correlation relationship with melt fluidity. As displayed in Figure 2b, the melt flow index of SAN, CPE and their blends were determined at 230 °C with an applied weight of 5.0 kg. The zero value indicates that the melt of polymer cannot be extruded from the die under the test conditions, meaning the processing fluidity of the CPE elastomer is extremely poor. As can be seen, the melt flow index decreases as the CPE content increases, namely, CPE has greatly reduced the melt fluidity of SAN/CPE blends and possibly increased the melt strength of the blends.

### 3.2. Compatibility of SAN/CPE Blends

**Figure 3:**
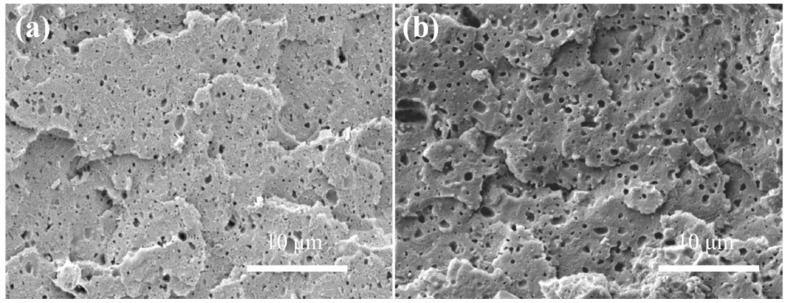
The fractured surface morphology of SAN/CPE blends with 10 wt.% (**a**) and 20 wt.% (**b**) CPE content. All blend samples for observation were quickly smashed after soaking in liquid nitrogen for 60 min.

The foaming process and foam morphology of polymer blends are affected by several factors, such as phase interface, crystallinity and melt rheological properties, which are related to the compatibility between two components. In this paper, the compatibility of SAN/CPE blends was investigated in relation to their microstructures and thermal properties. Figure 3a,b shows the SEM micrographs of the cryogenic fractured surface of SAN/CPE blends with 10 wt.% and 20 wt.% CPE elastomer content, respectively. As can be seen from the micrographs, the CPE particles as a dispersed phase were uniformly distributed in the SAN matrix, but the elastomer particles easily detached from the matrix to form holes when the blends were fractured in liquid nitrogen. These holes show clear and smooth interfaces, indicating poor interface bonding between components.

**Figure 4:**
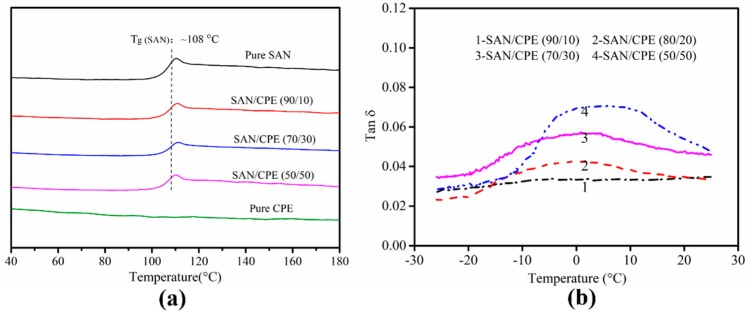
(**a**) DSC scanning curves of neat SAN, neat CPE and SAN/CPE blends; (**b**) The loss factor curves of SAN/CPE blends from DMA scanning.

As shown in Figure 4, in order to further determine the compatibility of SAN and CPE, the thermal and thermomechanical performances of the unfoamed samples were studied by DSC and DMA. The glass transition temperature (T_g_) of individual components in blends can be easily obtained from their scanning curves. The compatibility of components can be reliably assessed by changes in the glass transition temperature. If the blend has two glass transition temperatures, it is regarded as an incomplete compatible blend system. 

From the DSC scanning curves shown in Figure 4a, after eliminating the thermal history, glass transition occurs at about 108 °C in SAN resin and the glass transition temperature of SAN/CPE blends is almost the same as that of pure SAN. Although only one glass transition temperature was found in the curves, the blends could not be considered a compatible system. According to [25,26], chlorinated polyethylene has the same carbon chain backbone as polyethylene without unsaturated bonds, resulting in a low T_g_ far below room temperature, which is not conducive to measuring heat changes at low temperature by a DSC analyzer with high sensitivity to thermal changes. Thus, owing to the greater sensitivity to modulus change induced by molecular motion, a dynamic mechanical analysis method is often used to characterize multiple conformational transitions of polymers, and is especially suitable for characterization of multi-component systems. From the DMA scanning curves in Figure 4b, the tan δ versus temperature curves of the samples have a broad peak between −10 °C and 20 °C, suggesting that polymer chains have more intense molecular motion in this temperature range. The broad peak enlarges gradually with the increase in CPE content.

By considering the characteristics of each component in the blends, such as molecular structures, crystallization properties, thermal properties, etc., there is sufficient reason to believe that the broad peak in the temperature range of −10 to 20 °C was caused by internal loss due to the movement of CPE molecular chains in the blend. In other words, the CPE component in the blend experienced an obvious glass transition process in such a temperature range. Based on the DSC and DMA analysis, the SAN/CPE blend obviously had two glass transition temperatures. Therefore, it is easy to understand the incompatibility of SAN/CPE composites considering their independent glass transition temperature and poor interface bonding.

### 3.3. Porous Structures of Foamed SAN/CPE Samples

#### 3.3.1. Foaming Behavior of Neat SAN and Neat CPE

**Figure 5:**
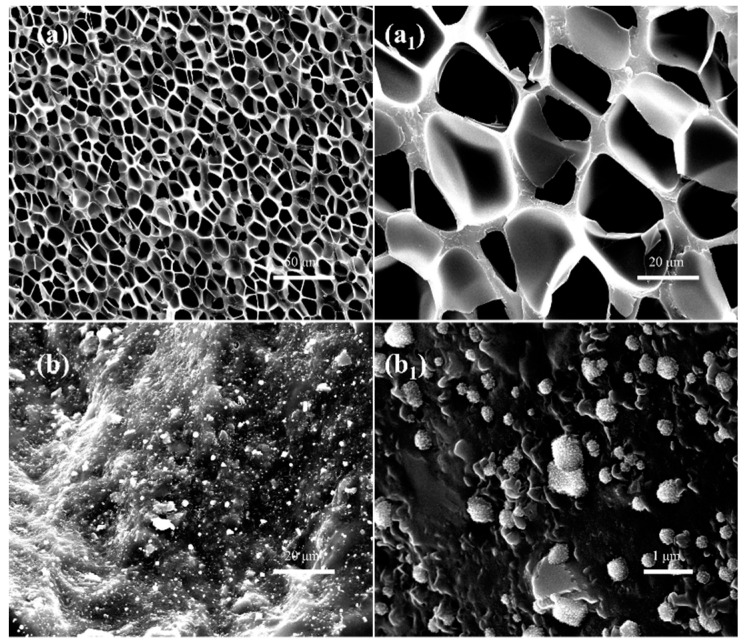
SEM images of neat SAN (**a**,**a_1_**) and neat CPE (**b**,**b_1_**) after rapid depressurization foaming treatment at 135 °C and 20 MPa.

The fractured surface morphologies of pure SAN and pure CPE after foaming treatment were investigated by scanning electron microscope. As displayed in Figure 5, the SAN sample was well foamed via rapid depressurization, while the CPE elastomer did not form pores under the same foaming conditions. 

As can be seen in Figure 5a, the SAN foam has dense micropores with an average cell diameter of about 9.6 µm and an average cell density of 8.4 × 10^8^ cells/cm^3^. The volume expansion ratio of SAN microcellular foam is about 1.52, that is, the corresponding apparent density of foam is about 0.657 g/cm^3^. Obviously, the foams have uniformly distributed pores. Since SAN resin is an amorphous copolymer with good processing fluidity and each part of such resin has similar adsorption patterns to CO_2_, which is conducive to forming a homogeneous gas/polymer saturation system, then, homogeneous nucleation is initiated and uniform bubbles develop. 

For CPE foams, only a few relevant studies on chemical foaming can be found in the literature at present, but very few are based on physical foaming. In the case of chemical foaming, the chemical blowing agents were first evenly mixed with chlorinated polyethylene, and then the mixture was heated at a suitable temperature higher than the decomposition temperature of the blowing agent, which generated large amounts of gas due to the decomposition of the reagent and resulted in the formation of numerous pores in the polymer matrix. However, SEM micrographs of the fractured surface showed a nonporous structure of CPE samples processed with supercritical carbon dioxide as the blowing agent, as illustrated in Figure 5b.

Obviously, although the CPE elastomer is an amorphous polymer, as evidenced by the DSC curves in Figure 4a, it cannot be foamed using supercritical CO_2_ foaming in current experimental conditions. As far as we know, chlorinated polyethylene is prepared by randomly replacing hydrogen atoms on polyethylene chains with chlorine atoms, which greatly enhances the molecular interaction and improves Mooney viscosity of the melt. In addition, the molecules may produce partial crosslinking during the substitution reaction. These factors are not conducive to the diffusion and adsorption of CO_2_ and inevitably affect the foaming behavior of chlorinated polyethylene.

It can easily be seen from high magnification SEM images that pure CPE actually still contains many tiny particles, as displayed in Figure 5b,b_1_. After eliminating the possibility of mixing impurities, these particles are most likely to be reinforcing fillers or crosslinked substances. These substances appear to have little effect on improving the foamability of the CPE matrix because several CPE samples in the same batch could not be foamed under various foaming conditions. The possible reason for this is that these particles, especially cross-linked substances, can affect the rheological properties of the matrix and greatly inhibit the growth of bubbles, resulting in the poor foamability of CPE matrix [27,28]. The specific composition of these particles, its role in the matrix, and effects on the adsorption of supercritical fluid will be examined in detail in future work.

#### 3.3.2. Microcellular Morphologies of SAN/CPE Blend Foams

**Figure 6:**
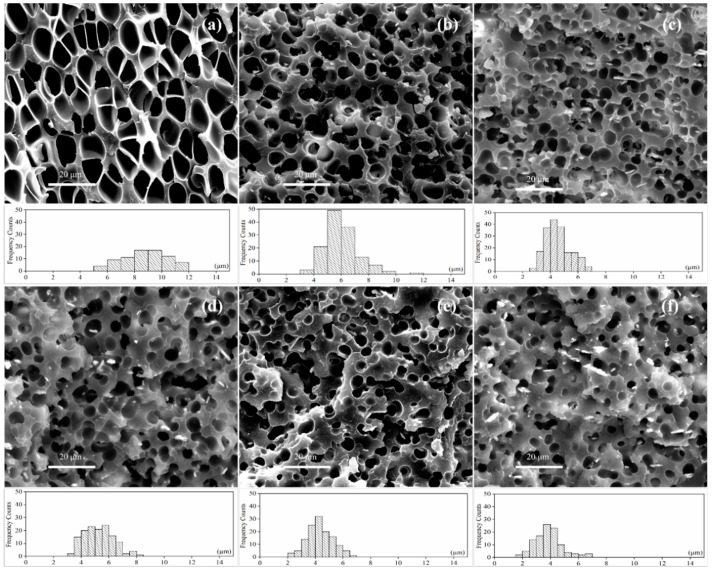
SEM micrographs of SAN/CPE foams. (**a**) Neat SAN; (**b**) 10 wt.% CPE; (**c**) 20 wt.% CPE; (**d**) 30 wt.% CPE; (**e**) 40 wt.% CPE; (**f**) 50 wt.% CPE. All samples were foamed at 130 °C and 25 MPa via rapid depressurization foaming and then they were quickly smashed after soaking in liquid nitrogen for 30 min in order to observe cross section morphology.

**Figure 7:**
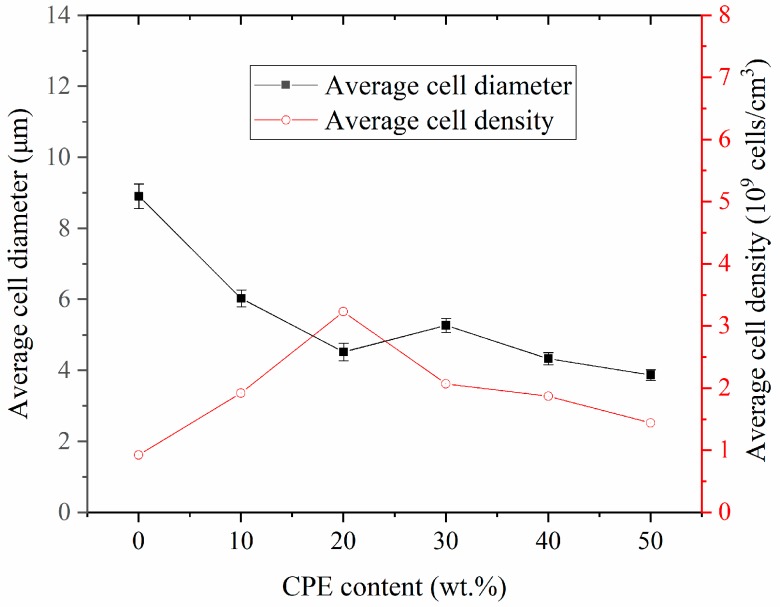
Average cell diameter and cell density of SAN/CPE composite foams.

As illustrated in Figure 6, the cellular morphologies of foamed SAN/CPE blends are very different from that of pure SAN, and the blend foams have unevenly distributed pores with thick pore walls as compared with SAN foams. The statistical results obtained from the SEM micrographs by image processing software are shown in Figure 7. The changes in average cell diameter and cell density with increasing CPE content are described by the black polyline and red polyline, respectively. As CPE content increased from 10–50 wt.% with an interval of 10 wt.%, the average pore diameter of their foams was 6.03, 4.52, 5.27, 4.33 and 3.87 µm, respectively, their corresponding average cell density was 1.92, 3.23, 2.07, 1.87 and 1.44 × 10^9^ cells/cm^3^ and the volume expansion ratios were about 1.23, 1.16, 1.18, 1.14 and 1.06, respectively. Apparently, the average pore diameter of SAN/CPE foams was smaller than SAN foam with an average pore size of 8.91 µm, however, their cell density was higher than 9.22 × 10^8^ cells/cm^3^ of pure SAN microcellular foam. These results indicate that the addition of chlorinated polyethylene elastomer significantly affected the foaming behavior and porous morphologies of the blends. In addition, the average pore diameter of the foamed sample with 30 wt.% CPE elastomer seems to be a bit higher and did not follow the trend, probably because some of pores were connected to be elongated pores with bigger average pore diameter value, as shown in the statistical histogram of Figure 6d. 

It was clearly observed that the pore size of blend foams decreases with increasing CPE content, with similar variation tendencies in the melt flow index for SAN/CPE composites. As described in the previous section, CPE elastomer is incompatible with SAN resin and could not be foamed using scCO_2_ as a physical blowing agent in this experiment. Therefore, the elastomer particles are reasonably considered to be organic fillers, which improved the melt viscoelasticity of blends and inhibited the growth of bubbles, which inevitably resulted in a decrease in average pore size.

Many factors can affect the cell density of polymer blend foam in the supercritical CO_2_ foaming process, such as melt rheological properties, foamability of components, equilibrium solubility and microphase structures of the blends. Since all samples, see Figure 6, were foamed in the same batch, the composition and phase structure of the blend are important factors affecting the cell density of these microcellular foams. Evidently, the proportion of SAN in the blend reduces as the nonfoamable component of CPE elastomer increases, while the cell density of SAN/CPE foams are larger than that of SAN foam. These facts indicated that bubble nucleation in the blend is different from the homogeneous nucleation of pure SAN during scCO_2_ foaming. For the incompatible SAN/CPE blends, there exists a large number of interfaces between two components and the foaming process has a higher bubble nucleation efficiency by heterogeneous nucleation [29,30,31]. 

As shown in Figure 7, the cell density increased significantly as the CPE content increased from 10 to 20 wt.%, while it decreased with the increase in CPE content from 30 to 50 wt.%. A possible reason is that power CPE granules were dispersed evenly into the matrix, which led to numerous phase interfaces for intensive heterogeneous nucleation during foaming. However, when CPE content increased further, many elastomer particles tended to melt into larger unfoamed regions and reduced phase interfaces in the blend, which led to less bubble nucleation at the interfaces, finally resulting in decreasing cell density in the blend foams with too much CPE elastomer. Nonetheless, all SAN/CPE microcellular foams have higher cell densities than pure SAN foams, which is most likely due to the interfaces’ heterogeneous nucleation of bubbles.

In order to investigate the influence of foaming process parameters on morphologies, several samples of the SAN/CPE (80/20) blend were foamed at temperatures of 70, 90, 110 and 130 °C, with a saturation pressure of 15 MPa. The average cell diameter and cell density of SAN/CPE foams as illustrated in Table 1, were obtained from the SEM images of the fractured sections by image processing software. From the statistical results, the average cell diameter of the foams increased gradually with the increase in temperature. The trend was similar to the foaming process of a single component amorphous polymer. Simultaneously, the cell density also increases slightly with increased saturation temperature. The possible reason could be related to the good adsorption of carbon dioxide in CPE component at high saturation temperatures, which is conducive to generating high cell density. 

Specifically, the foamed sample with 20 wt.% CPE had an average pore size of 4.02 μm and pore density of 2.07 × 10^9^ cells/cm^3^ under the foaming conditions of 130 °C and 15 MPa, which is smaller than 4.52 μm and 3.23 × 10^9^ cells/cm^3^ at 130 °C and 25 MPa. It is clear that high saturated pressure with supercritical carbon dioxide is beneficial in obtaining greater bubble density [22]. Because a greater pressure difference can be created by rapid depressurizing from a higher saturation pressure, this leads to dense bubble nucleation by intense thermodynamic fluctuations. On the other hand, the average cell diameter slightly increased by increasing the saturated pressure, Similar phenomenon has also been seen in other published literature [32]. The most likely reason is that the great expansion force from the large pressure drop makes pores grow bigger by overcoming the imposed constraints from the unfoamed content during the curing process.

#### 3.3.3. Interconnected Porous Structures in SAN/CPE Foams

**Figure 8:**
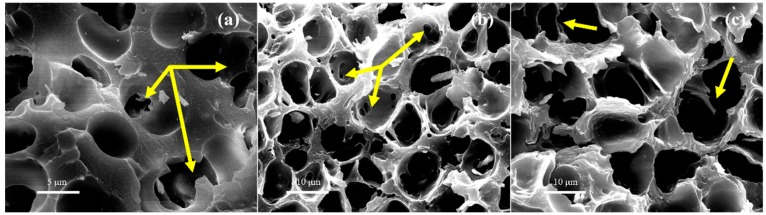
The interconnected pores in SAN/CPE foams. (**a**) 10 wt.% CPE; (**b**) 20 wt.% CPE; (**c**) 30 wt.% CPE; which sample **a** was foamed at 130 °C and 25 MPa and sample **b** and **c** were foamed at 110 °C and 20 MPa.

There is an interesting phenomenon that many interconnected pores, as indicated by the arrows in Figure 8, were generated in SAN/CPE foams by scCO_2_ foaming. As can also be seen in Figure 8, several adjacent micropores were connected by tiny holes in thick pore walls, which is very different from pure SAN microcellular foam with closed-pores. The open porosity of SAN/CPE foams with 10, 20 and 30 wt.% CPE were determined by an automatic density analyzer ULTRAPYC 1200e with argon as a medium and the results were 19.73, 30.61 and 33.76% respectively, which are significantly higher than that of SAN microcellular foam with 5.31% open porosity. These results indicate that partly interconnected pores were formed inside the SAN/CPE microcellular foams via scCO_2_ foaming. 

**Figure 9:**
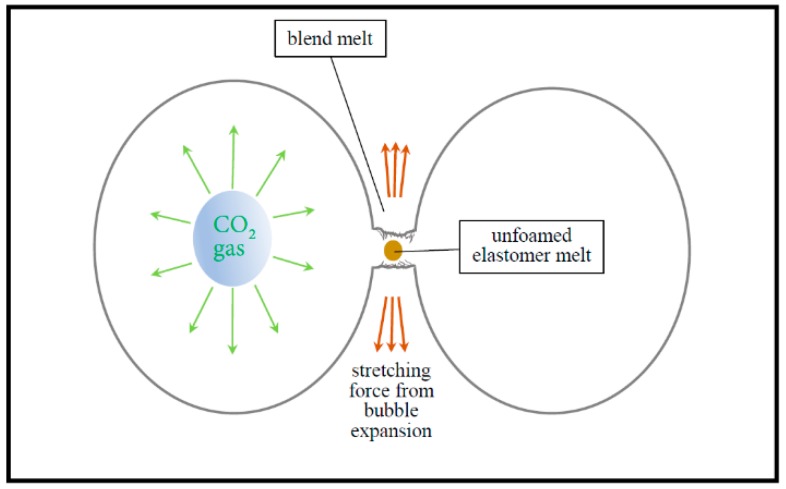
Schematic diagram of the pore opening mechanism during the foaming process of incompatible SAN/CPE blends.

Usually, it is difficult to fabricate open-pore SAN foams without using pore opener, even under extreme foaming conditions with high foaming temperature and saturated pressure. Also, it is often necessary to use porogens such as surfactant and soluble salt to assist in obtaining interconnected porous structures [33,34]. However, there were no soluble particles or any other processing aids in the preparation of SAN/CPE blends. Thus, it is possible that such blend foams with open-pores involve a different pore-opening mechanism, which this is described by the schematic diagram in Figure 9. According to classical foaming theory [35], bubbles begin to grow quickly after rapid depressurization, which causes the melt surrounding expanding bubbles to withstand tensile forces. For a single component system with insufficient melt strength, as bubbles grow further, the melt walls separating bubbles become thinner and thinner until they eventually rupture into open-pores. However, for a multicomponent system, the complex influencing factors of pore opening are still related to compatibility, melt rheological properties and the foamability of individual components. As previously stated, the SAN/CPE blend has a large number of phase interfaces with weak interactions due to poor compatibility, and two components of the blend have quite different melt flow characteristics. In addition, the elastomer component in the blend cannot be foamed under a foaming process based on the one-step depressurization method. Thus, the blend melt wall is subjected to stretching and ruptures due to the weak phase interfaces and further grows into interconnected tiny pores connecting adjacent micropores.

It is clear that such pore opening phenomenon in an incompatible blend of non-crosslinked plastic and elastomer is related to the composition and bubble growth process. The melt walls with few interfaces are difficult to rupture when the dosage of CPE is too small. Additionally, too much CPE will also be conducive to pores opening due to its difficulty in foaming. In this experiment, it was found that the uniformly dispersed CPE with 20 to 30 wt.% content and sufficient bubble growth were favorable for the formation of interconnected porous structures in SAN/CPE foams.

### 3.4. Mechanical Properties of SAN/CPE Microcellular Foams

**Figure 10:**
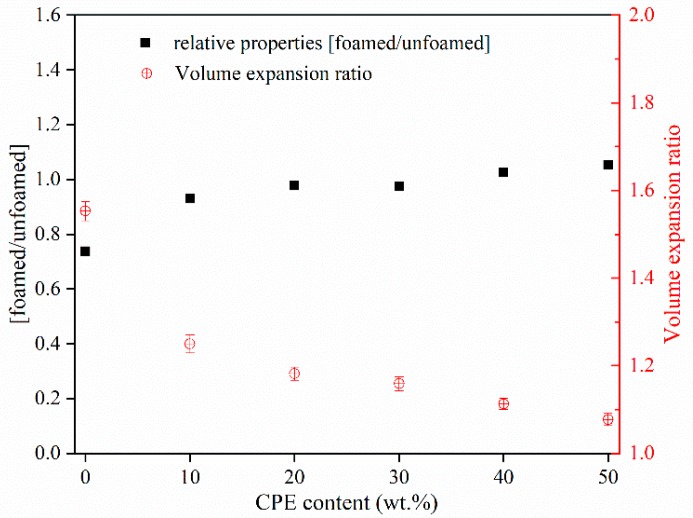
Relative properties for the foamed samples of SAN and SAN/CPE blends.

The impact properties of foamed samples with notched geometry were tested in the Charpy configuration. The relative property index, the impact strength ratio of foamed and unfoamed samples, was used to describe the impact property changes in the foamed sample versus the unfoamed sample [36]. As displayed in Figure 10, the impact strength of SAN foam with 1.55 times volume expansion was much lower than its unfoamed sample with an impact strength about 1.87 KJ/m^2^. This is probably due to the reduction in solid skeleton withstand impact per unit volume. In general, poor compatibility of the blend and the increased apparent density and cell size of foam [37] damages the mechanical resistivity. However, the impact properties SAN/CPE foams were close to the corresponding unfoamed samples, as showed in Figure 10. The possible explanation is that the CPE component was able to increase the apparent density and reduce the cell size of the foam, resulting in improving the impact resistance of SAN/CPE foam. In addition, the CPE elastomer with good impact toughness can withstand most of the impact energy when subjected to impact, which is also beneficial to good mechanical performances.

## 4. Conclusions

In this study, a series of SAN/CPE microcellular foams were fabricated by rapid depressurization with supercritical carbon dioxide as the physical blowing agent. The effects of CPE elastomer on the foaming process and cellular morphologies of SAN/CPE blends were systematically investigated. It was found that although CPE elastomer could not be foamed in this experiment, it contributed to a smaller pore size and a larger cell density in the blend foams as compared to pure SAN foam under the same foaming conditions. The possible reason is that the elastomer, with almost zero melt flow index, greatly reduced the melt flowability of the blend and inhibited the growth of gaseous bubbles, resulting in smaller pores and thicker pore walls. Also, numerous phase interfaces inside the blend were conducive to increased cell density due to efficient bubble heterogeneous nucleation during foaming. Moreover, many interconnected pores existed in the SAN/CPE foams, which was possibly induced by pore walls rupturing from phase interfaces with weak interaction. Evidently, the foaming process and cellular morphologies of polymer blends can be controlled by modulating the composition, compatibility, melt rheological properties and foamability of individual components of the blend. This study also presents an effective means for preparing polymeric microcellular foams with interconnected pores, which can be used as filtration for separation or water treatment.

## Figures and Tables

**Table 1:** Average cell diameter and cell density of SAN/CPE foams with 20 wt.% CPE at different temperature with same soaking pressure of 15 MPa.

Temperature (°C)	Average Cell Diameter (μm)	Cell Density (Cells/cm^3^)
**70**	2.31 ± 0.16	1.32 × 10^9^
**90**	3.67 ± 0.09	1.52 × 10^9^
**110**	3.83 ± 0.12	1.68 × 10^9^
**130**	4.02 ± 0.11	2.07 × 10^9^
